# ENNET: inferring large gene regulatory networks from expression data using gradient boosting

**DOI:** 10.1186/1752-0509-7-106

**Published:** 2013-10-22

**Authors:** Janusz Sławek, Tomasz Arodź

**Affiliations:** 1 Department of Computer Science, Virginia Commonwealth University, Richmond, Virginia

**Keywords:** Gene regulatory networks, Network inference, Ensemble learning, Boosting

## Abstract

**Background:**

The regulation of gene expression by transcription factors is a key determinant of cellular phenotypes. Deciphering genome-wide networks that capture which transcription factors regulate which genes is one of the major efforts towards understanding and accurate modeling of living systems. However, reverse-engineering the network from gene expression profiles remains a challenge, because the data are noisy, high dimensional and sparse, and the regulation is often obscured by indirect connections.

**Results:**

We introduce a gene regulatory network inference algorithm ENNET, which reverse-engineers networks of transcriptional regulation from a variety of expression profiles with a superior accuracy compared to the state-of-the-art methods. The proposed method relies on the boosting of regression stumps combined with a relative variable importance measure for the initial scoring of transcription factors with respect to each gene. Then, we propose a technique for using a distribution of the initial scores and information about knockouts to refine the predictions. We evaluated the proposed method on the DREAM3, DREAM4 and DREAM5 data sets and achieved higher accuracy than the winners of those competitions and other established methods.

**Conclusions:**

Superior accuracy achieved on the three different benchmark data sets shows that ENNET is a top contender in the task of network inference. It is a versatile method that uses information about which gene was knocked-out in which experiment if it is available, but remains the top performer even without such information. ENNET is available for download from https://github.com/slawekj/ennet under the GNU GPLv3 license.

## Background

Regulation of gene expression is a key driver of adaptation of living systems to changes in the environment and to external stimuli. Abnormalities in this highly coordinated process underlie many pathologies. At the transcription level, the control of the amount of mRNA transcripts involves epigenetic factors such as DNA methylation and, in eukaryotes, chromatin remodeling. But the key role in both prokaryotes and eukaryotes is played by transcription factors (TF), that is, proteins that can bind to DNA in the regulatory regions of specific genes and act as repressors or inducers of their expression. Many interactions between transcription factors and genes they regulate have been discovered through traditional molecular biology experiments. With the introduction of high-throughput experimental techniques for measuring gene expression, such as DNA microarrays and RNA-Seq, the goal moved to reverse-engineering genome-wide gene regulatory networks (GRNs) [1]. Knowledge of GRNs can facilitate finding mechanistic hypotheses about differences between phenotypes and sources of pathologies, and can help in the drug discovery and bioengineering.

High throughput techniques allow for collecting genome-wide snapshots of gene expression across different experiments, such as diverse treatments or other perturbations to cells [2]. Analyzing these data to infer the regulatory network is one of the key challenges in the computational systems biology. The difficulty of this task arises from the nature of the data: they are typically noisy, high dimensional, and sparse [3]. Moreover, discovering direct causal relationships between genes in the presence of multiple indirect ones is not a trivial task given the limited number of knockouts and other controlled experiments. Attempts to solve this problem are motivated from a variety of different perspectives. Most existing computational methods are examples of influence modeling, where the expression of a target transcript is modeled as a function of the expression levels of some selected transcription factors. Such a model does not aim to describe physical interactions between molecules, but instead uses inductive reasoning to find a network of dependencies that could explain the regularities observed among the expression data. In other words, it does not explain mechanistically how transcription factors interact with regulated genes, but indicate candidate interactions with a strong evidence in expression data. This knowledge is crucial to prioritize detailed studies of the mechanics of the transcriptional regulation.

One group of existing methods describes GRN as a system of ordinary differential equations. The rate of change in expression of a transcript is given by a function of the concentration levels of transcription factors that regulate it. Network inference includes two steps: a selection of a model and an estimation of its parameters. Popular models imply linear functions a priori [4-7]. Bayesian Best Subset Regression (BBSR) [8] has been proposed as a novel model selection approach, which uses Bayesian Information Criterion (BIC) to select an optimal model for each target gene. Another group of methods employ probabilistic graphical models that analyze multivariate joint probability distributions over the observations, usually with the use of Bayesian Networks (BN) [9-11], or Markov Networks (MN) [12]. Various heuristic search schemes have been proposed in order to find parameters of the model, such as greedy-hill climbing or the Markov Chain Monte Carlo approach [13]. However, because learning optimal Bayesian networks from expression data is computationally intensive, it remains impractical for genome-wide networks.

Other approaches are motivated from statistics and information theory. TwixTwir [14] uses double two-way *t*-test to score transcriptional regulations. The null-mutant z-score algorithm [15] scores interactions based on a z-score transformed knockout expression matrix. Various algorithms rely on estimating and analyzing cross-correlation and mutual information (MI) of gene expression in order to construct a GRN [16-20], including ANOVA *η*^2^ method [21]. Improvements aimed at removing indirect edges from triples of genes have been proposed, including techniques such as the Data Processing Inequality in ARACNE [22,23], and the adaptive background correction in CLR [24]. Another method, NARROMI [25], eliminates redundant interactions from the MI matrix by applying ODE-based recursive optimization, which involves solving a standard linear programming model.

Recently, machine-learning theory has been used to formulate the network inference problem as a series of supervised gene selection procedures, where each gene in turn is designated as the target output. One example is MRNET [26], which applies the maximum relevance/minimum redundancy (MRMR) [27] principle to rank the set of transcription factors according to the difference between mutual information with the target transcript (maximum relevance) and the average mutual information with all the previously ranked transcription factors (minimum redundancy). GENIE3 [28] employs Random Forest algorithm to score important transcription factors, utilizing the embedded relative importance measure of input variables as a ranking criterion. TIGRESS [29] follows a similar approach but is based on the least angle regression (LARS). Recently, boosting [30,31] was also used to score the importance of transcription factors, in ADANET [32] and OKVAR-Boost [33] methods.

In this paper, we propose a method that combines gradient boosting with regression stumps, augmented with statistical re-estimation procedures for prioritizing a selected subset of edges based on results from the machine-learning models. We evaluated our method on the DREAM3, DREAM4 and DREAM5 network inference data sets, and achieved results that in all cases were better than the currently available methods.

## Methods

### The ENNET algorithm

#### Formulating the gene network inference problem

The proposed algorithm returns a directed graph of regulatory interactions between *P* genes in form of a weighted adjacency matrix $V\in {\mathbb{R}}^{P\times P}$, where *v*_*i*,*j*_ represents regulation of gene *j* by gene *i*. As an input, it takes gene expression data from a set of experiments, together with the meta-data describing the conditions of the experiments, including which genes were knocked out. Usually, the raw expression data need to be pre-processed before any inference method could be applied to reverse-engineer a GRN. Pre-processing has a range of meanings, here it is regarded as a process of reducing variations or artifacts, which are not of the biological origin. It is especially important when the expression is measured with multiple high-density microarrays [34]. Concentration levels of transcripts must be adjusted and the entire distribution of adjusted values aligned with a normal distribution. Methods for normalization of expression data are outside of the scope of our work. The data we used were already normalized using RMA [34,35] by the DREAM challenge organizers. We further normalized the expression data to zero mean and unit standard deviation.

The network inference process relies heavily on the type of expression data provided as an input. Two main groups of expression profiles are: the one with known, and the one with unknown initial perturbation state of the expression of genes in the underlying network of regulatory interactions. For example, knockout and knockdown data are provided with the additional meta-data, which describe which genes were initially perturbed in each experiment. On the other hand, multifactorial and time series data are usually expression profiles of an unknown initial state of genes. Wildtype, knockout, knockdown, and multifactorial data describe the expression of initially perturbed genes, which are however in a steady state at the time of measurement, whereas time series data describe the dynamics of the expression levels of initially perturbed genes. The types of data available in popular benchmark data sets are summarized in Table 1.

**Table 1:** Different types of expression data provided in popular data sets

**Data set**	**WT**	**KO**	**KD**	**MF**	**TS**
DREAM3 size 100	•	•	•	◦	•
DREAM4 size 100	•	•	•	◦	•
DREAM4 size 100 MF	◦	◦	◦	•	◦
DREAM5^⋆^	•	•	•	•	•

Different types of expression data provided in popular data sets: WT- Wildtype, KO- Knockouts, KD- Knockdowns, MF- Multifactorial, TS- Time series, • Available, ◦ Unavailable. ⋆) Even though all the data types are available, they are all processed as MF.

The variability of possible input scenarios poses a problem of representing and analyzing expression data. Here, we operate on an *N*×*P* expression matrix *E*, where *e*_*i*,*j*_ is the expression value of the j-th gene in the i-th sample. Columns of matrix *E* correspond to genes, rows correspond to experiments. We also define a binary perturbation matrix *K*, where *k*_*i*,*j*_ is a binary value corresponding to the j-th gene in the i-th sample, just like in the matrix *E*. If *k*_*i*,*j*_ is equal to 1, it means that the j-th gene is known to be initially perturbed, for example knocked out, in the i-th experiment. Otherwise *k*_*i*,*j*_ is equal to 0. If no information is available about knockouts, all values are set to 0.

#### Decomposing the inference problem into gene selection problems

We decompose the problem of inferring the network of regulatory interactions targeting all *P* genes into *P* independent subproblems. In each subproblem incoming edges from transcription factors to a single gene transcript are discovered. For the *k*-th decomposed subproblem we create a target expression vector *Y*_*k*_ and a feature expression matrix *X*_−*k*_. Columns of the *X*_−*k*_ matrix constitute a set of possible transcription factors. Vector *Y*_*k*_ corresponds to the expression of the transcript, which is possibly regulated by transcription factors from *X*_−*k*_. In a single gene selection problem we decide which TFs contribute to the target gene expression across all the valid experiments. Columns of *X*_−*k*_ correspond to all the possible TFs, but if a target gene k is also a transcription factor, it is excluded from *X*_−*k*_. We do not consider a situation in which a transcription factor would have a regulatory interaction with itself. When building the target vector *Y*_*k*_ corresponding to the k-th target gene, *k*∈{1,...,*P*}, we consider all the experiments valid except from the ones in which the k-th gene was initially perturbed, as specified in the perturbation matrix *K*. We reason that the expression value of the k-th gene in those experiments is not determined by its TFs, but by the external perturbation. Each row in the *Y*_*k*_ vector is aligned with a corresponding row in the *X*_−*k*_ matrix. In order to justify all the possible interactions we need to solve a gene selection problem for each target gene. For example, if a regulatory network consists of four genes (*P*=4), we need to solve four gene selection problems. In the k-th problem, *k*∈{1,2,3,4}, we find which TFs regulate the k-th target gene. In other words, we calculate the k-th column of the output adjacency matrix *V*.

#### Solving the gene selection problems

Once the target gene expression vector *Y*_*k*_ and the TF expression matrix *X*_−*k*_ are created for each gene *k*, we solve each *k*-th gene selection problem independently, in the following way. We search for the subset of columns in *X*_−*k*_ that are related to the target vector *Y*_*k*_ by an unknown function *f*_*k*_, as shown in Equation 1,

(1) $$\forall k\in \{1,\mathrm{...},P\},\;\exists \;f_{k}:Y_{k}=f_{k}(X_{-k})+\varepsilon_{k},$$

where *ε*_*k*_ is a random noise. A function *f*_*k*_ represents a pattern of regulatory interactions that drive the expression of the k-th gene. We want *f*_*k*_ to rely only on a small number of genes acting as transcription factors, those that are the true regulators of gene *k*. Essentially, this is a feature selection or a gene selection task [28,32,36,37], where the goal is to model the target response *Y*_*k*_ with an optimal small set of important predictor variables, i.e., a subset of columns of the *X*_−*k*_ matrix. A more relaxed objective of the gene selection is the variable ranking, where the relative relevance for all input columns of the *X*_−*k*_ matrix is obtained with respect to the target vector *Y*_*k*_. The higher a specific column is in that ranking, the higher the confidence that a corresponding TF is in a regulatory interaction with the target gene *k*.

**Figure 1:**
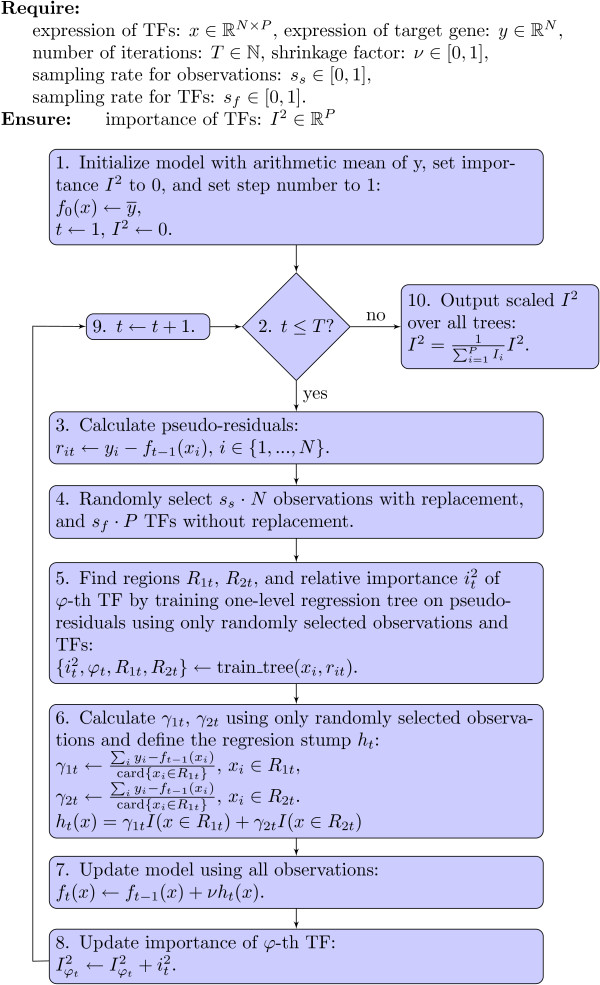
**The flowchart of the ENNET algorithm.** ENNET algorithm is a modification of a Gradient Boosting Machine algorithm, with a squared error loss function and a regression stump base learner. The algorithm calculates a vector of importance scores of transcription factors, which can possibly regulate a target gene. It is invoked *P* times in a problem of inferring a *P*-gene network, i.e., a *P*-column adjacency matrix *V*.

Our solution to the variable ranking involves ensemble learning. We use an iterative regression method, which in each iteration chooses one transcription factor based on an optimality criterion, and adds it to the non-linear regression ensemble. The main body of our method, presented in Figure 1, is based on Gradient Boosting Machine [38] with a squared error loss function. First, ENNET initializes *f*_0_ to be an optimal constant model, without selecting any transcription factor. In other words, *f*_0_ is initialized to an average of *Y*_*k*_. At each next t-th step the algorithm creates an updated model *f*_*t*_, by fitting a base learner *h*_*t*_ and adding it to the previous model *f*_*t*−1_. The base learner is fitted to a sample of pseudo residuals, with respect to a sample of transcription factors, and thus is expected to reduce the error of the model. Pseudo-residuals are re-calculated at the beginning of each iteration with respect to the current approximation *f*_*t*_. As a base learner, we use regression stumps, which select a single TF that best fits pseudo residuals. A regression stump *h*_*t*_(*x*) partitions the expression values *x* of a candidate TF into two disjoint regions *R*_1*t*_ and *R*_2*t*_, where $R_{2t}=\mathbb{R}-R_{1t}$, and returns values *γ*_1*t*_ and *γ*_2*t*_, respectively, for those regions, as shown in Equation 2,

(2) $$h_{t}(x)=\gamma_{1t}I(x\in R_{1t})+\gamma_{2t}I(x\in R_{2t}),$$

where *I* is the identity function returning the numerical 1 for the logical true, and the numerical 0 for the logical false. Regions *R*_1*t*_, *R*_2*t*_ are induced such that the least-squares improvement criterion is maximized:

(3) $$i^{2}(R_{1t},R_{2t})=\frac{w_{1t}w_{2t}}{w_{1t}+w_{2t}}{\left(\gamma_{1t}-\gamma_{2t}\right)}^{2},$$

where *w*_1*t*_, *w*_2*t*_ are proportional to the number of observations in regions *R*_1*t*_, *R*_2*t*_ respectively, and *γ*_1*t*_, *γ*_2*t*_ are corresponding response means. That is, *γ*_1*t*_ is the average of the values from the vector of pseudo-residuals for those samples where an expression of the chosen TF falls into the region *R*_1*t*_. The value of *γ*_2*t*_ is defined in an analogous way. The averages *γ*_1*t*_ and *γ*_2*t*_ are used as the regression output values for regions *R*_1*t*_ and *R*_2*t*_, respectively, as shown in Equation 2. The criterion in Equation 3 is evaluated for each TF, and the transcription factor with the highest improvement is selected. In each t-th step, we only use a random portion of rows and columns of *X*_−*k*_, sampled according to the observation sampling rate *s*_*s*_, and the TF sampling rate *s*_*f*_.

The procedure outlined above creates a non-linear regression model of the target gene expression based on the expression of transcription factors. However, in the network inference, we are interested not in the regression model as a whole, but only in the selected transcription factors. In each t-th step of the ENNET algorithm, only one TF is selected as the optimal predictor. The details of the regression model can be used to rank the selected TFs by their importance. Specifically, if a transcription factor *φ*_*t*_ is selected in an iteration *t*, an improvement $i_{t}^{2}$ serves as an importance score $I_{\varphi_{t}}^{2}$ for that *φ*_*t*_-th TF. If the same TF is selected multiple times at different iterations, its final importance score is a sum of the individual scores.

In the training of the regression model, the parameter *ν*, known as a shrinkage factor, is used to scale a contribution of each tree by a factor *ν*∈(0,1) when it is added to the current approximation. In other words, *ν* controls the learning rate of the boosting procedure. Shrinkage techniques are also commonly used in neural networks. Smaller values of *ν* result in a larger training risk for the same number of iterations *T*. However, it has been found [38] that smaller values of *ν* reduce the test error, and require correspondingly larger values of *T*, which results in a higher computational overhead. There is a trade-off between these two parameters.

#### Refining the inferred network

Once the solutions of the independent gene selection problems are calculated, we compose the adjacency matrix *V* representing a graph of inferred regulatory interactions. Each of the solutions constitutes a single column-vector, therefore we obtain the adjacency matrix *V* by binding all the partial solutions column-wise. Then we apply a re-evaluation algorithm to achieve an improved final result. The first step does not require any additional data to operate other than the previously calculated adjacency matrix *V*. It exploits the variance of edge probabilities in the rows of *V*, i.e., edges outgoing from a single transcription factor, as a measure of the effect of transcriptional regulation. We score transcription factors based on their effects on multiple targets. We assume that the effect of transcriptional regulation on a directly regulated transcript is stronger than the one of the regulation on indirectly regulated transcripts, e.g. transcripts regulated through another transcription factor. Otherwise, knocking out a single gene in a strongly connected component in a network of regulatory interactions would cause the same rate of perturbation of the expression level of all the transcripts in that component. As a measure of that effect we use previously a calculated adjacency matrix *V* and multiply each row of *V* matrix by its variance $\sigma_{i}^{2}$. An updated adjacency matrix *V*^1^ is given by Equation 4:

(4) $$\forall (i,j):v_{i,j}^{1}=\sigma_{i}^{2}\cdot v_{i,j},$$

where $\sigma_{i}^{2}$ is a variance in the i-th row of *V*. Note that *V* matrix is built column-wise, i.e., a single column of *V* contains the relative importance scores of all the transcription factors averaged over all the base learners with respect to a single target transcript. On the other hand, rows of *V* matrix are calculated independently in different subproblems of the proposed inference method. Each row of *V* contains relative importance scores with respect to a different target transcript. We reason that if a transcription factor regulates many target transcripts, e.g. a transcription factor is a hub node, the variance in a row of *V* corresponding to that transcription factor is elevated and therefore it indicates an important transcription factor.

The second step of refining the network requires knockout expression data. We reason that direct regulation of a transcript by a transcription factor would lead to a distinct signature in the expression data if the transcription factor was knocked out. A similar reasoning gave foundations for the null-mutant z-score method [15] of reverse-engineering GRNs. However, in the proposed method this step is only applied if knockout expression profiles are available. In this step we calculate an adjacency matrix *V*^2^, which is an update to an already derived adjacency matrix *V*^1^, as shown in Equation 5:

(5) $$\begin{array}{l} \forall (i,j):v_{i,j}^{2}=|\frac{\bar{e_{\alpha (i),j}}-\bar{e_{\beta (i),j}}}{\sigma_{j}}|\cdot v_{i,j}^{1},\\ \alpha (i)=\{r:k_{r,i}\neq 0\},\beta (i)=\{r:k_{r,i}=0\}, \end{array}$$

where $\bar{e_{\alpha (i),j}}$ is an average expression value of the j-th transcript in all the experiments *α*(*i*) in which the i-th gene was knocked-out, as defined by *K* matrix, $\bar{e_{\beta (i),j}}$ is the mean expression value for that transcript across all the other knockout experiments, *β*(*i*), and *σ*_*j*_ is the standard deviation of the expression value of that transcript in all the knockout experiments. The $\left|\frac{\bar{e_{\alpha (i),j}}-\bar{e_{\beta (i),j}}}{\sigma_{j}}\right|$ coefficient shows how many standard deviations the typical expression of the j-th transcript was different from the average expression in the experiment in which its potential i-th transcription factor was knocked-out.

### Performance evaluation

A considerable attention has been devoted in recent years to the problem of evaluating performance of the inference methods on adequate benchmarks [35,39]. The most popular benchmarks are derived from well-studied *in vivo* networks of model organisms, such as *E. coli*[40] and *S. cerevisiae*[41], as well as artificially simulated *in silico* networks [39,42-45]. The main disadvantage of *in vivo* benchmark networks is the fact that experimentally confirmed pathways can never be assumed complete, regardless of how well the model organism is studied. Such networks are assembled from known transcriptional interactions with strong experimental support. As a consequence, gold standard networks are expected to have few false positives. However, they contain only a subset of the true interactions, i.e., they are likely to contain many false negatives. For this reason, artificially simulated *in silico* networks are most commonly used to evaluate network inference methods. Simulators [39] mimic real biological systems in terms of topological properties observed in biological *in vivo* networks, such as modularity [46] and occurrences of network motifs [47]. They are also endowed with dynamical models of a transcriptional regulation, thanks to the use of non-linear differential equations and other approaches [42,48,49], and consider both transcription and translation processes in their dynamical models [48-50] using a thermodynamic approach. Expression data can be generated deterministically or stochastically and experimental noise, such as the one observed in microarrays, can be added [51].

Here, we used several popular benchmark GRNs to evaluate the accuracy of our proposed algorithm and compare it with the other inference methods. The data sets we used come from Dialogue for Reverse Engineering Assessments and Methods (DREAM) challenges and are summarized in Table 1. We evaluated the accuracy of the methods using the Overall Score metric proposed by the authors of DREAM challenges [35], as shown in Equation 6:

(6) $$\text{Overall Score}=-\frac{1}{2}\cdot \log_{10}({\bar{p}}_{\text{aupr}}\cdot {\bar{p}}_{\text{auroc}}),$$

where ${\bar{p}}_{\text{aupr}}$ and ${\bar{p}}_{\text{auroc}}$ are geometric means of p-values of networks constituting each DREAM challenge, relating to an area under the Precision-Recall curve (AUPR) and an area under the ROC curve (AUROC), respectively.

## Results and discussion

**Figure 2:**
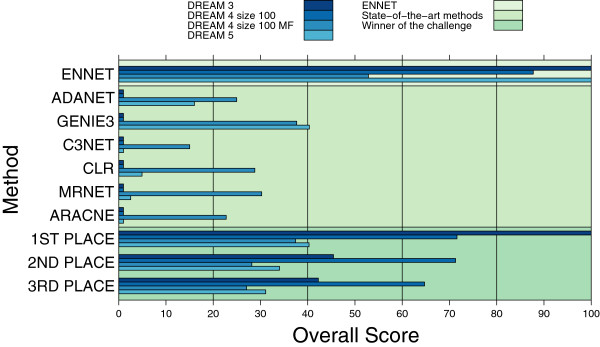
**The Overall Score of GRN inference methods by data set.** Results of the different inference methods on DREAM challenges. Results of the state-of-the-art methods were collected after running the algorithms with the default sets of parameters on pre-processed data. Results in the “Winner of the challenge” part of the figure correspond to the best methods participating in the challenge.

We assessed the performance of the proposed inference algorithm on large, universally recognized benchmark networks of 100 and more genes, and compared it to the state-of-the-art methods. We summarize the results of running different inference methods in Figure 2. For a comparison we selected a range of established methods from literature: ARACNE, CLR, and MRNET as implemented in the *minet* R package [52], GENIE3 and C3NET as implemented by their respective authors, our previously reported method ADANET, and the top three performers in each of the three DREAM challenges as listed on the DREAM web site. Some of the methods were designed for use with knockout data, while others are developed with multifactorial data in mind, where no information is given about the nature of the perturbations. Therefore, depending on the nature of the particular DREAM data set, only the suitable group of methods is used for the comparison.

### The accuracy of ENNET

DREAM3 [15,53,54] features *in silico* networks and expression data simulated using GeneNetWeaver software. Benchmark networks were derived as subnetworks of a system of regulatory interactions from known model organisms: *E. coli* and *S. cerevisiae*. In this study we focus on a DREAM3 size 100 subchallenge, as the largest of DREAM3 suite. The results of all the competing methods except those that are aimed at multifactorial problems are summarized in Table 2. ENNET and Yip et al. methods achieved the best Overall Scores for that subchallenge, as well as the best scores for all the individual networks. However, it is believed from the analysis of the later challenges [39] that Yip et al. method made a strong assumption on the Gaussian type of a measurement noise, which was used in DREAM3, but was no longer used in later DREAM challenges. For example, in DREAM4 challenge Yip et al. method was ranked 7th.

**Table 2:** Results of the different inference methods on DREAM3 networks, challenge size 100

**Method**	**Network (AUPR/AUROC respectively)**										**Overall**
	**1**		**2**		**3**		**4**		**5**		
**Experimental results**											
ENNET	0.627	0.901	**0.865**	**0.963**	**0.568**	0.892	**0.522**	0.842	0.384	0.765	>**300**
Winner of the challenge											
Yip et al.	**0.694**	**0.948**	0.806	0.960	0.493	**0.915**	0.469	**0.856**	**0.433**	**0.783**	>**300**
2nd	0.209	0.854	0.249	0.845	0.184	0.783	0.192	0.750	0.161	0.667	45.443
3nd	0.132	0.835	0.154	0.879	0.189	0.839	0.179	0.738	0.164	0.667	42.240

Results of the different inference methods on DREAM3 networks, challenge size 100. An area under the ROC curve (AUROC) and an area under the Precision-Recall curve (AUPR) are given for each network respectively. The overall Score for all the networks is given in the last column. The best results for each column are in bold. Numbers in the “Experimental results” part of the table were collected after running the algorithms with the default sets of parameters on pre-processed data. However, ADANET, GENIE3, CLR, C3NET, MRNET, and ARACNE methods, as they are originally defined, take a multifactorial matrix as an input, which is unavailable in this challenge. Therefore they were excluded from the comparison. Numbers in the “Winner of the challenge” part of the table correspond to the best methods participating in the challenge.

DREAM4 challenge [15,53,54] was posted one year after DREAM3 challenge. It features two large subchallenges: DREAM4 size 100, and DREAM4 size 100 multifactorial. For each subchallenge, the topology of the benchmark networks were derived from the transcriptional regulatory system of *E. coli* and *S. cerevisiae*. In DREAM4 size 100 subchallenge all the data types listed in Table 1 were available except multifactorial, therefore ADANET, GENIE3, CLR, C3NET, MRNET, and ARACNE methods were excluded from the comparison. The results of all the methods are summarized in Table 3. ENNET method clearly outperformed all the others and achieved consistently high scores across all the benchmark networks. In the second DREAM4 large subchallenge, DREAM4 size 100 multifactorial, only multifactorial data were available, therefore all the methods were included in the comparison, and run as originally designed. The results of all the methods are summarized in Table 4. ENNET achieved the best Overall Score.

**Table 3:** Results of the different inference methods on DREAM4 networks, challenge size 100

**Method**	**Network (AUPR/AUROC respectively)**										**Overall**
	**1**		**2**		**3**		**4**		**5**		
**Experimental results**											
ENNET	**0.604**	0.893	**0.456**	**0.856**	**0.421**	**0.865**	**0.506**	**0.878**	**0.264**	**0.828**	**87.738**
Winner of the challenge											
Pinna et al.	0.536	**0.914**	0.377	0.801	0.390	0.833	0.349	0.842	0.213	0.759	71.589
2nd	0.512	0.908	0.396	0.797	0.380	0.829	0.372	0.844	0.178	0.763	71.297
3rd	0.490	0.870	0.327	0.773	0.326	0.844	0.400	0.827	0.159	0.758	64.715

Results of the different inference methods on DREAM4 networks, challenge size 100. An area under the ROC curve (AUROC) and an area under the Precision-Recall curve (AUPR) are given for each network respectively. The Overall Score for all the networks is given in the last column. The best results for each column are in bold. Numbers in the “Experimental results” part of the table were collected after running the algorithms with the default sets of parameters on pre-processed data. However, ADANET, GENIE3, CLR, C3NET, MRNET, and ARACNE methods, as they are originally defined, take a multifactorial matrix as an input, which is unavailable in this challenge. Therefore they were excluded from the comparison. Numbers in the “Winner of the challenge” part of the table correspond to the best methods participating in the challenge.

**Table 4:** Results of the different inference methods on DREAM4 networks, challenge size 100 multifactorial

**Method**	**Network (AUPR/AUROC respectively)**										**Overall**
	**1**		**2**		**3**		**4**		**5**		
**Experimental results**											
ENNET	**0.184**	0.731	**0.261**	**0.807**	**0.289**	**0.813**	**0.291**	**0.822**	**0.286**	**0.829**	**52.839**
ADANET	0.149	0.664	0.094	0.605	0.191	0.703	0.172	0.712	0.182	0.694	24.970
GENIE3	0.158	**0.747**	0.154	0.726	0.232	0.777	0.210	0.795	0.204	0.792	37.669
C3NET	0.077	0.562	0.095	0.588	0.126	0.621	0.113	0.687	0.110	0.607	15.015
CLR	0.142	0.695	0.118	0.700	0.178	0.746	0.174	0.748	0.174	0.722	28.806
MRNET	0.138	0.679	0.128	0.698	0.204	0.755	0.178	0.748	0.187	0.725	30.259
ARACNE	0.123	0.606	0.102	0.603	0.192	0.686	0.159	0.713	0.166	0.659	22.744
Winner of the challenge											
GENIE3	0.154	0.745	0.155	0.733	0.231	0.775	0.208	0.791	0.197	0.798	37.428
2nd	0.108	0.739	0.147	0.694	0.185	0.748	0.161	0.736	0.111	0.745	28.165
3rd	0.140	0.658	0.098	0.626	0.215	0.717	0.201	0.693	0.194	0.719	27.053

Results of the different inference methods on DREAM4 networks, challenge size 100 multifactorial. An area under the ROC curve (AUROC) and an area under the Precision-Recall curve (AUPR) are given for each network respectively. The Overall Score for all the networks is given in the last column. The best results for each column are in bold. Numbers in the “Experimental results” part of the table were collected after running the algorithms with the default sets of parameters on pre-processed data. Numbers in the “Winner of competition” part of the table correspond to the best methods participating in the challenge.

Three benchmark networks in DREAM5 [35] were different in size, and structured with respect to different model organisms. However, this time expression data of the only one network were simulated *in silico*, the two other sets of expression data were measured in real experiments *in vivo*. Like in all DREAM challenges, *in silico* expression data were simulated using an open-source GeneNetWeaver simulator [54]. However, DREAM5 was the first challenge where participants were asked to infer GRNs on a genomic scale, e.g. for thousands of target genes, and hundreds of known transcription factors. Gold standard networks were obtained from two sources: RegulonDB database [40], and Gene Ontology (GO) annotations [55]. The results of all the inference methods for DREAM5 expression data are summarized in Table 5. ENNET achieved the best score for the *in silico* network, and the best Overall Score, as well as the best individual AUROC scores for all the networks. Clearly all the participating methods achieved better scores for an *in silico* network than for either one of *in vivo* networks. ENNET shows better *in vivo* results than the other methods in terms of an area under the the ROC curve. Still, predictions for *in vivo* expression profiles show a low overall accuracy. One of the reasons for a poor performance of the inference methods for such expression profiles is a fact that experimentally confirmed pathways, and consequently gold standards derived from them, cannot be assumed complete, regardless of how well is a model organism known. Additionally, there are regulators of gene expression other than transcription factors, such as miRNA, and siRNA. As shown in this study, *in silico* expression profiles provide enough information to confidently reverse-engineer their underlying structure, whereas *in vivo* data hide a much more complex system of regulatory interactions.

**Table 5:** Results of the different inference methods on DREAM5 networks

**Method**	**Network (AUPR/AUROC respectively)**						**Overall**
	**1**		**3**		**4**		
**Experimental results**							
ENNET	**0.432**	**0.867**	0.069	**0.642**	0.021	**0.532**	>**300**
ADANET	0.261	0.725	0.083	0.596	0.021	0.517	16.006
GENIE3	0.291	0.814	**0.094**	0.619	0.021	0.517	40.335
C3NET	0.080	0.529	0.026	0.506	0.018	0.501	0.000
CLR	0.217	0.666	0.050	0.538	0.019	0.505	4.928
MRNET	0.194	0.668	0.041	0.525	0.018	0.501	2.534
ARACNE	0.099	0.545	0.029	0.512	0.017	0.500	0.000
Winner of the challenge							
GENIE3	0.291	0.815	0.093	0.617	0.021	0.518	40.279
ANOVA *η*^2^	0.245	0.780	0.119	0.671	**0.022**	0.519	34.023
TIGRESS	0.301	0.782	0.069	0.595	0.020	0.517	31.099

Results of the different inference methods on DREAM5 networks. An area under the ROC curve (AUROC) and an area under the Precision-Recall curve (AUPR) are given for each network respectively. The Overall Score for all the networks is given in the last column. The best results for each column are in bold. Numbers in the “Experimental results” part of the table were collected after running the algorithms with the default sets of parameters on pre-processed data. Numbers in the “Winner of the challenge” part of the table correspond to the best methods participating in the challenge.

### Computational complexity of ENNET

Computational complexity of ENNET depends mainly on the computational complexity of the regression stump base learner, which is used in the main loop of the algorithm. As shown in Figure 1, we call the regression stump algorithm *T* times for each k-th target gene, *k*∈{1,...,*P*}. Given a sorted input, a regression stump is *O*(*PN*) complex. We sort the expression matrix in an *O*(*PN* log*N*) time. All the other instructions in the main loop of ENNET are at most *O*(*N*). The computational complexity of the whole method is thus *O*(*PN* log*N*+*TP*^2^*N*+*TPN*). Because, in practice, the dominating part of the sum is *TP*^2^*N*, we report a final computational complexity of ENNET as *O*(*TP*^2^*N*), and compare it to the other inference methods in Table 6. Note that the measure for the information-theoretic methods: CLR, MRNET, and ARACNE does not include a calculation of the mutual information matrix.

**Table 6:** The computational complexity of ENNET and the other GRN inference methods

**Method**	**Complexity**
ENNET	*O*(*TP*^2^*N*), *T*=5000
ADANET	*O*(*CTP*^2^*N*), *C*=30, $T=\lceil \sqrt{P}\rceil$
GENIE3	*O*(*TKPN* log*N*), *T*=1000, $K=\lceil \sqrt{P}\rceil$
C3NET	*O*(*P*^2^)
CLR	*O*(*P*^2^)
MRNET	*O*(*fP*^2^), *f*∈[1,*P*]
ARACNE	*O*(*P*^3^)

The computational complexity of ENNET and the other GRN inference methods with respect to the number of genes *P* and the number of samples *N*. The computational complexity of CLR, MRNET, and ARACNE is given without calculating the Mutual Information matrix.

When implementing ENNET algorithm we took advantage of the fact that gene selection problems are independent of each other. Our implementation of the algorithm is able to calculate them in parallel if multiple processing units are available. User can choose from variety of parallel backends including multicore package for a single computer and parallelization based on Message Passing Interface for a cluster of computers. The biggest data we provided as input in our tests were *in vivo* expression profiles of *S. cerevisiae* from the DREAM 5 challenge. These are genome-wide expression profiles of 5950 genes (333 of them are known transcription factors) measured in 536 experiments. It took 113 minutes and 30 seconds to calculate the network on a standard desktop workstation with one Intel®;Core™i7-870 processor with 4 cores and two threads per core (in total 8 logical processors) and 16 GB RAM. However, it took only 16 minutes and 40 seconds to calculate the same network on a machine with four AMD Opteron™6282 SE processors, each with 8 cores and two threads per core (in total 64 logical processors) and 256 GB RAM. All the data sets from the DREAM 3 and the DREAM 4 challenges were considerably smaller, up to 100 genes. It took less than one minute to calculate each of these networks on a desktop machine.

### Setting parameters of ENNET

The ENNET algorithm is controlled by four parameters: the two sampling rates *s*_*s*_ and *s*_*f*_, the number of iterations *T* and the learning rate *ν*. The sampling rate of samples *s*_*s*_ and the sampling rate of transcription factors *s*_*f*_ govern the level of randomness when selecting, respectively, rows and columns of the expression matrix to fit a regression model. The default choice of the value of *s*_*s*_ is 1, i.e., we select with replacement a bootstrap sample of observations of the same size as an original training set at each iteration. Because some observations are selected more than once, around 0.37 of random training samples are out of bag in each iteration. It is more difficult to choose an optimal value of *s*_*f*_, which governs how many transcription factors are used to fit each base learner. Setting this parameter to a low value forces ENNET to score transcription factors, even if their improvement criterion, as shown in Equation 2, would not have promoted them in a pure greedy search, i.e., *s*_*f*_=1. However, if a chance of selecting a true transcription factor as a feature is too low, ENNET will suffer from selecting random genes as true regulators.

**Figure 3:**
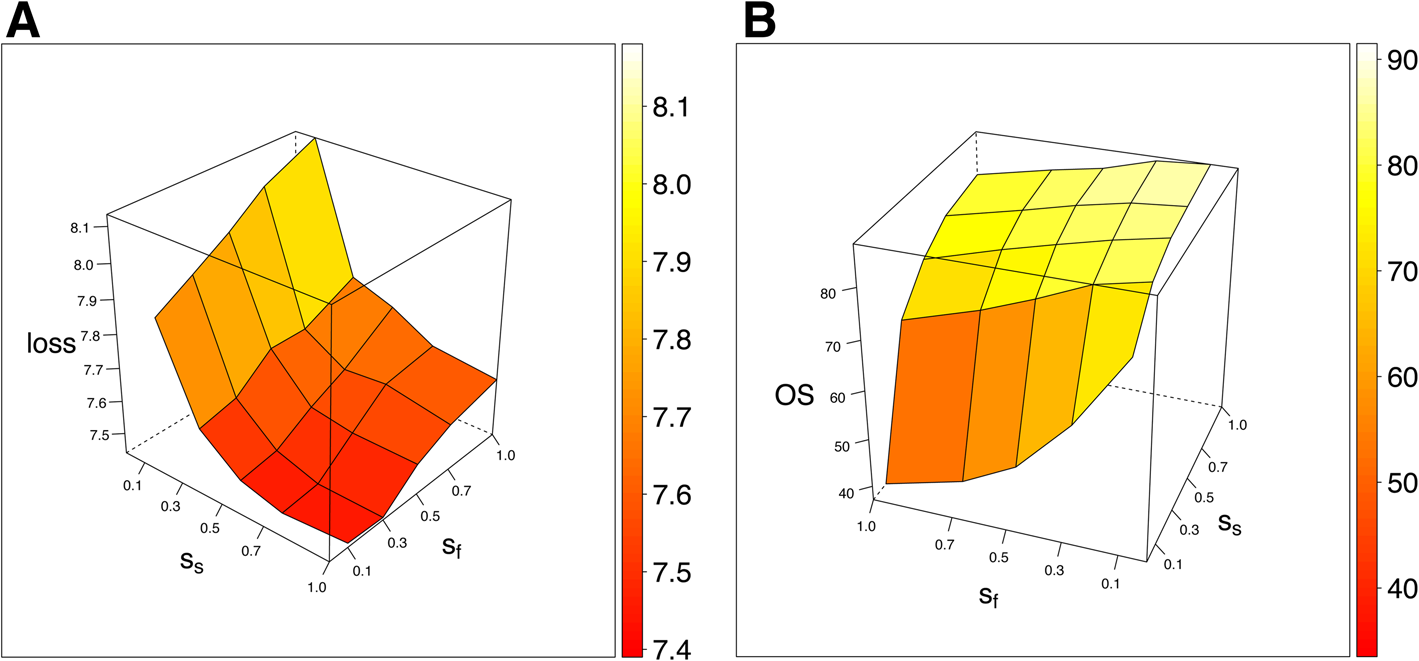
**The analysis of the sampling rates *s***_***s***_** and *s***_***f ***_** for DREAM 4 size 100 challenge: the Overall Score and a loss.** The analysis of the sampling rates *s*_*s*_ and *s*_*f*_ for the DREAM 4 size 100 challenge. **A:** For each set of parameters (*s*_*s*_, *s*_*f*_, *M*, *ν*) ∈ {0.1,0.3,0.5,0.7,1} × {0.1,0.3,0.5,0.7,1} × {5000} × {0.001} we analyzed an average 5-fold cross-validated loss over all the observations (across all gene selection problems) from all 5 networks. The minimal average loss was achieved for high values of *s*_*s*_ = 1 and low values of *s*_*f*_ = 0.3. **B:** We also compared the measure based on an average loss with the original Overall Score, as proposed by the authors of the DREAM challenge. The results were consistent across the two measures, i.e., the parameters that gave low average loss also led to accurate network predictions (a high Overall Score).

Even though reverse-engineering of GRNs does not explicitly target a problem of predicting gene expression, we choose the values of sampling rates such that the squared-error loss of a prediction of the target gene expression as given by *f*_*T*_ (see Figure 1) is minimal. This is done without looking at the ground truth of regulatory connections. For each benchmark challenge we performed a grid search over (*s*_*s*_,*s*_*f*_)∈{0.1,0.3,0.5,0.7,1}×{0.1,0.3,0.5,0.7,1} with fixed *ν*=0.001, *T*=5000. For each specific set of parameters we analyzed an average 5-fold cross-validated loss over all the observations (across all gene selection problems). We further analyze our approach with respect to one of the challenges: DREAM4 size 100, as shown in Figure 3. The minimal average loss was achieved for *s*_*s*_=1 and *s*_*f*_=0.3 (see Figure 3 A), which is consistent with the default parameters proposed for Random Forest algorithm [28]. We also compared the measure based on an average loss with the Overall Score as defined by Equation 6. The results were consistent across the two measures, i.e., a selection of parameters that gave a low average loss also led to the accurate network predictions (see Figure 3 B). An advantage of the average loss measure is a fact that the gold standard network is not used to tune parameters.

**Figure 4:**
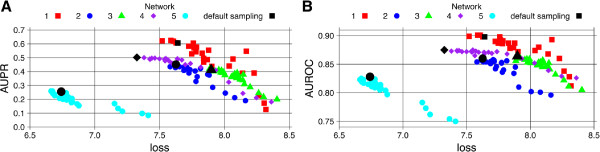
**The analysis of the sampling rates *s***_***s ***_**and *s***_***f***_** for DREAM 4 size 100 challenge: AUPR, AUROC, and a loss.** The analysis of the sampling rates *s*_*s*_ and *s*_*f*_ for DREAM 4 size 100 challenge. **A:** For each set of parameters (*s*_*s*_, *s*_*f*_, *M*, *ν*) ∈ {0.1,0.3,0.5,0.7,1} × {0.1,0.3,0.5,0.7,1} × {5000} × {0.001} we analyzed an area under the Precision-Recall curve (AUPR) in function of an average 5-fold cross-validated loss over all the observations (across all gene selection problems) from all 5 networks. For each network AUPR is decreasing in a function of a loss. For each network a point corresponding to the default set of parameters is highlighted, i.e., (*s*_*s*_, *s*_*f*_, *M*, *ν*) = (1,0.3,5000,0.001). Usually, the default set of parameters gives the minimal loss (maximal AUPR). **B:** By analogy, different choices of parameters lead to a different area under the ROC curve (AUROC). The two measures are consistent with each other.

In Figure 4 we present a detailed analysis of the accuracy of the GRN inference across different networks of the DREAM4 size 100 challenge. Each point on both Figure 4 A and Figure 4 B is a result of running ENNET with different parameters: (*s*_*s*_,*s*_*f*_)∈{0.1,0.3,0.5,0.7,1}×{0.1,0.3,0.5,0.7,1} with fixed *ν*=0.001, *T*=5000. The highlighted points are corresponding to *s*_*s*_=1, *s*_*f*_=0.3, *ν*=0.001, *T*=5000. An area under the Precision-Recall curve and an area under the ROC curve are two different measures of the accuracy of an inferred network, which are well preserved across the five networks: for each separate network we observe that AUPR and AUROC decreases in a function of an average loss. As the Overall Score is closely related to AUPR and AUROC, the results shown in Figure 4 explain the shape of a surface shown in Figure 3.

**Figure 5:**
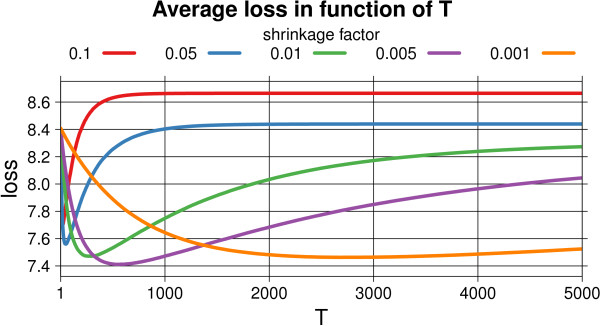
**The analysis of the number of iterations T and the shrinkage *ν *for the DREAM 4 size 100 challenge.** The analysis of the number of iterations T and the shrinkage factor *ν* for DREAM 4 size 100 challenge. These two parameters are closely coupled: the lower is the shrinkage parameter *ν*, the more iterations *T* are needed to train the model such that it achieves the minimal loss.

As ENNET uses boosting, it needs a careful tuning of the number of iterations *T* and the learning rate *ν*. It has been shown [38] that parameters *T* and *ν* are closely coupled. Usually the best prediction results are achieved when *ν* is fixed to a small positive number, e.g. *ν*≤0.001, and the optimal value of *T*Y is found in a process of cross-validation. As described above, we reason that the choice of parameters, which gives a low average loss on a cross-validated test set, leads to an accurate network prediction. Therefore in Figure 5 we present how an average loss depends on *T*∈{1,...,5000} for different values of *ν*∈{0.001,0.005,0.01,0.05,0.1}, with fixed *s*_*s*_=1, *s*_*f*_=0.3. Each of the line shows how much ENNET overtrains the data for a given *T* and *ν*. Finally, the optimal choice of parameters for DREAM4 size 100 challenge is *s*_*s*_=1, *s*_*f*_=0.3, *T*=5000, *ν*=0.001. Following the same practice, we used this default set of parameters: *s*_*s*_=1, *s*_*f*_=0.3, *T*=5000, *ν*=0.001 to evaluate ENNET algorithm on all the benchmark networks using ground truth, i.e., for calculating the Overall Score and comparing it to the other algorithms.

### Stability of ENNET

Because ENNET uses random sampling of samples and features at each iteration of the main loop, as shown in Figure 1, it may calculate two different networks for two different executions on the same expression data. With the default choice of parameters, i.e., *s*_*s*_=1, *s*_*f*_=0.3, *T*=5000, *ν*=0.001, we expect numerous random resamplings, and therefore we need to know if a GRN calculated by ENNET is stable between different executions. We applied ENNET to the 5 networks that form DREAM 4 size 100 benchmark, repeating the inference calculations independently ten times for each network. Then, for each network, we calculated a Spearman’s rank correlation between all pairs among the ten independent runs. The lowest correlation coefficient we obtained was *ρ*>0.975, with *p*-value <2.2*e*−16, indicating that the networks that result from independent runs are very similar. This proves that ENNET, despite being a randomized algorithm, finds a stable solution to the inference problem.

## Conclusions

We have proposed the ENNET algorithm for reverse-engineering of Gene Regulatory Networks. ENNET uses a variety of types of expression data as an input, and shows robust performance across different benchmark networks. Moreover, it does not assume any specific model of a regulatory interaction and do not require fine-tuning of its parameters, i.e., we define the default set of parameters, which promises accurate predictions for the future networks. Nevertheless, together with the algorithm, we propose a procedure of tuning parameters of ENNET towards minimizing empirical loss. Processing genome-scale expression profiles is feasible with ENNET: including up to a few hundred transcription factors, and up to a few thousand regulated genes. As shown in this study, the proposed method compares favorably to the state-of-the-art algorithms on the universally recognized benchmark data sets.

## Competing interests

The authors declare that they have no competing interests.

## Authors’ contributions

JS and TA conceived the method and drafted the manuscript. JS implemented the method and ran the experiments. JS and TA read and approved the final manuscript.
